# Roles of mir155hg and TNF-α in evaluation of prognosis of patients with systemic lupus erythematosus

**DOI:** 10.5937/jomb0-45870

**Published:** 2024-04-23

**Authors:** Xiaojing Gu, Hu Chen, Rongping Li, Dibin Guo

**Affiliations:** 1 The First Affiliated Hospital of Gannan Medical College, Department of Rheumatology and Immunology, Ganzhou, China

**Keywords:** systemic lupus erythematosus, MIR155HG, TNF-α, sistemski eritematozni lupus, MIR155HG, TNF-α

## Abstract

**Background:**

Systemic lupus erythematosus (SLE) is a chronic inflammatory autoimmune disease characterized by multi-organ multi-system inflammation, causing severe damage to various organs or systems. Recent studies have shown that miR-155 can affect the progression of Lupus Nephritis via regulating TNF-a. The present study aims to explore the roles of MIR155HG and TNF-a in the evaluation of prognosis of patients with SLE, so as to provide a basis for clinical work.

**Methods:**

A total of 130 patients with SLE admitted to our hospital were selected, were selected from June 2015 to December 2017., and the SLE disease activity index (SLEDAI) score was given. The expressions of MIR155HG and TNF-a were detected via quantitative reverse transcription-polymerase chain reaction (qRT-PCR), the incidence of complications during treatment was observed, and the associations of MIR155HG and TNF-a with SLEDAI before treatment and complications were analyzed. All patients were followed up after discharge, and the related factors to the prognosis of patients were analyzed via Cox regression analysis.

**Results:**

The levels of MIR155HG and TNF-a were higher in patients with an SLEDAI score of 10-14 points than those in patients with an SLEDAI score of 5-9 points and 0-4 points. MIR155HG and TNF-a were positively correlated with the incidence of infection, renal damage and cardiac damage (r=0.623, 0.533 and 0.621; r=0.431, 0.498 and 0.552) (P<0.05). Moreover, there was also a positive correlation (r=0.3398, P<0.001) between the expressions of serum MIR155HG and TNF-a in SLE patients. SLEDAI score ≥10 points, complications during hospitalization, and highly-expressed MIR155HG and TNFa were risk factors related to the prognosis of patients.

**Conclusions:**

MIR155HG and TNF-a affect the activity of SLE, and the high expressions of them promote the occurrence of such complications as infection, renal damage and cardiac damage, harming the prognosis.

## Abstract (sr)

**Uvod:**

Sistemski eritematozni lupus (SLE) je hronična inflamatorna autoimuna bolest koju karakteriše višeorganska multisistemska upala, koja izaziva ozbiljna oštećenja različitih organa ili sistema. Nedavne studije su pokazale da miR-155 može uticati na napredovanje lupus nefritisa regulacijom TNF-a. Ova studija ima za cilj da istraži uloge MIR155HG i TNF-a u proceni prognoze pacijenata sa SLE, kako bi se pružila osnova za klinički rad.

**Metode:**

Odabrano je ukupno 130 pacijenata sa SLE primljenim u našu bolnicu, odabrano od juna 2015. do decembra 2017. godine i dat je skor indeksa aktivnosti SLE bolesti (SLEDAI). Ekspresije MIR155HG i TNF-a su detektovane kvantitativnom reverznom transkripcijom-polimerazom lančanom reakcijom (qRT-PCR), uočena je učestalost komplikacija tokom lečenja, a analizirane su asocijacije MIR155HG i TNF-a sa SLEDAI pre tretmana i komplikacija. Svi pacijenti su praćeni nakon otpusta, a faktori povezani sa prognozom pacijenata analizirani su Cox regresionom analizom.

**Rezultati:**

Nivoi MIR155HG i TNF-a bili su viši kod pacijenata sa SLEDAI skorom od 10-14 poena nego kod pacijenata sa SLEDAI skorom od 5-9 poena i 0-4 poena. MIR155HG i TNF-a su bili u pozitivnoj korelaciji sa incidencom infekcije, oštećenja bubrega i oštećenja srca (r=0,623, 0,533 i 0,621; r=0,431, 0,498 i 0,552) (P<0,05). Štaviše, postojala je i pozitivna korelacija (r=0,3398, P<0,001) izme u ekspresije serumskog MIR155HG i TNF-a kod pacijenata sa SLE. SLEDAI skor ≥10 poena, komplikacije tokom hospitalizacije i visoko eksprimirani MIR155HG i TNF-a bili su faktori rizika koji se odnose na prognozu pacijenata.

**Zaključak:**

MIR155HG i TNF-a utiču na aktivnost SLE, a njihova visoka ekspresija promoviše nastanak takvih komplikacija kao što su infekcija, oštećenje bubrega i srčana oštećenja, što šteti prognozi.

## Introduction

Systemic lupus erythematosus (SLE) is a chronic inflammatory autoimmune disease characterized by multi-organ multi-system inflammation, causing severe damage to various organs or systems [1]. At the initial onset, SLE has complex and diverse manifestations, a long course of disease and complicated conditions. With the prolongation of disease course, the prognosis of patients is seriously affected [2].

Long non-coding ribonucleic acids (lncRNAs) are a class of non-coding RNAs with more than 200 nucleotides in length, and they were originally considered as »junk sequences« of genome transcription, without biological functions. In recent years, there is increasing evidence that lncRNAs play an important role in regulating the differentiation and maturation of immune cells, and they are important molecules involved in immunity and inflammation and also key players in autoimmune diseases such as SLE and rheumatoid arthritis. Moreover, lncRNAs are expected to become biomarkers for diagnosis and prognosis of disease [3]. MiR-155 host gene (MIR155HG), also known as B-cell integration cluster gene BIC, is an lncRNA with 1500 bp in length, named after the host gene of miR-155 [4] [5]. A study has found that MIR155HG is highly expressed in a variety of tumors, and its expression is up-regulated through the transcriptional activation of Myeloblastosis oncogene MYB, thereby leading to the down-regulation of many tumor suppressor genes [6]. It has been shown that the increased expression of miR-155 in peripheral blood mononuclear cells is a potential biomarker for lupus nephritis [7].

As a pleiotropic cytokine, tumor necrosis factor-α (TNF-α) can serve as not only a pro-inflammatory factor that mediates inflammatory cascade, causing tissue damage, but also an immunoregulatory factor that plays an important role in keeping the body's immune homeostasis [8] [9]. According to many studies, TNF-α/TNFRs signal excursion is one of the immunopathological foundations for the genetic susceptibility of SLE, the complexity of immune system disorders and the diversity of clinical manifestations [9]. Furthermore, literature has reported that the LncRNA-HOTAIR promotes the production of TNF-α in LPS-induced septicemic mouse myocardial cells by activating the NF-kB pathway [10]. Recent studies have shown that miR-155 can affect the progression of Lupus Nephritis via regulating TNF-α [11]. The present study aims to explore the roles of MIR155HG and TNF-α in the evaluation of prognosis of patients with SLE, so as to provide a basis for clinical work.

## Materials and methods

### General data and inclusion and exclusion criteria

A total of 130 patients with SLE admitted to our hospital, were selected from June 2015 to December 2017. Inclusion criteria: 1) Patients meeting the revised 1997 American College of Rheumatology diagnostic criteria for systemic lupus erythematosus (SLE) [12], 2) Those with initial episode of disease in the active phase, and 3) All patients voluntarily participated in the study in accordance with the principles of medical ethics. The study was approved by the respective hospital and conducted in compliance with ethical requirements.

Exclusion criteria: 1) patients with other connective tissue diseases, 2) those aged <20 years old, 3) those with recurrent SLE, 4) those complicated with severe organ dysfunction, severe infections or cardiovascular diseases, 5) those who could not cooperate due to nerve system diseases, or 6) those with incomplete medical data.

### Treatment methods

All patients underwent hormone therapy. Prednisone Acetate Tablets were orally taken (1 mg/kg×d) in the morning. The dose of drug was reduced at a rate of 5 mg/2 weeks as appropriate according to the changes in patients' conditions, and the initial dose was restored after impact therapy. 

### Observation indexes

Prior to treatment, 5 mL of fasting venous blood was drawn in the morning, and centrifuged at 3,000 rpm for 10 min. The serum was separated and stored at -70°C for later use. At the same time, the SLE disease activity index (SLEDAI) score was given: 0–4 points: basically no disease activity, 5–9 points: mild activity, 10–14 points: moderate activity, 15 points: severe activity. Based on the SLEDAI score, the patients were grouped, and the levels of serum MIR155HG and TNF-α were compared in different groups. 

### Quantitative reverse transcription-polymerase chain reaction (qRT-PCR)

The total RNA was extracted using TRIzol (Invitrogen, USA), and 2 mg of total RNA was synthesized into complementary deoxyribonucleic acid (cDNA) in a 20 μL system according to the instructions of the AMV RT kit (Takara, Dalian). RT-PCR was performed using 2×SYBR Green PCR Master Mix (Takara, Dalian) on a qPCR instrument. Then the corresponding forward and reverse primers (0.4 μmol/L) were designed and synthesized by Takara (Dalian) according to the target genes, followed by PCR amplification in a 15 μL system with cDNA as a template, and 3 parallel samples for each sample. The primers were as follows: MIR155HGF: 5'-GAGTGCT-GAAGGCTTGCTGT-3', R 5'-TTGAACATCCCAGT-GACCAG-3'. TNF-α F: 5'-GTGACAAGCCTGTAGCC-CAT-3', R: 5'-TATCTCTCAGCTCCACCCCA-3'. β-actin F: 5'-TCACCCACACTGTGCCCA-TCTACGA-3', R: 5'-CAGCGGAACCGCTCATTGCCAATGG-3'. The PCR conditions were as follows: 95 for 10 min, (95 for 15 s, 60 for 30 s, 72 for 30 s) × 40 cycles. The data obtained in 3 independent assays were analyzed using the formula RQ= 2^-**Δ**Δ**** Ct^.

### Laboratory examination

The anti-double-stranded DNA antibody (dsDNA antibody) was detected via indirect immunofluorescence, and the anti-extractable nuclear antigen (ENA) antibodies, including Smith (Sm)antibody, U1-RNP antibody, anti-nucleosome antibody (AnuA) and anti-ribosomal P protein (P-Prot) antibody, were detected via Western blotting using the kits provided by the Beijing Office of HUMAN GmbH. The incidence of complications during treatment was recorded. Infection: definite clinical symptoms and signs (body temperature >37°C), blood routine examination (WBC >10×10^9^/L), positive results of etiological examination, and effective antibiotic/antiviral treatment. Blood system damage: hemolytic anemia, leukopenia (<3.0×10^9^/L), lymphopenia (<1.1×10^9^/L), or thrombocytopenia (<100×10^9^/L). Renal damage: 24 h urinary protein quantification >0.5 g, or cast (erythrocyte, hemoglobin, granular or mixed cast), or/and lupus nephritis confirmed by renal biopsy. Central nervous system damage: based on the American College of Rheumatology nomenclature for neuropsychiatric lupus in 1999 [13]. Pulmonary damage: pulmonary interstitial changes, diffuse alveolar hemorrhage, and pulmonary arterial hypertension (diagnostic criteria: pulmonary arterial systolic pressure detected via color Doppler echocardiography in a resting state 4 kPa, excluding a history of rheumatic heart disease, myocardial infarction, chronic obstructive pulmonary disease and lung cancer), excluding other causes of lung lesions. Cardiac damage: myocarditis, cardiac insufficiency and arrhythmia, excluding other causes of heart disease.

### Follow-up

All patients were followed up after discharge via outpatient service or telephone. The endpoint was 5 years after follow-up or death of patients, during the follow-up period, 15 patients passed away.

### Statistical analysis

Statistic Package for Social Science (SPSS) 20.0 software (IBM, Armonk, NY, USA) was used for data analysis. Measurement data were expressed as mean ± standard deviation, and t test or one-way analysis of variance was used for the comparison. Spearman rank correlation analysis was adopted for the correlation analysis among different indexes. The association between MIR155HG and TNF-α was analyzed via Spearman linear correlation analysis. COX proportional hazards regression model was used for the prognostic analysis of patients. *P*<0.05 was considered to be statistically significant.

## Results

### Comparison of levels of MIR155HG and TNF-α in patients with different SLEDAI scores

The SLEDAI score was 0-4, 5-9, 10-14 and 15 points, respectively, in 66 cases, 35 cases, 19 cases and 10 cases. The results of qRT-PCR showed that the levels of serum MIR155HG and TNF-α had statistically significant differences (*P*<0.05). The levels of MIR155HG and TNF-α significantly rose in patients with a high SLEDAI score (*P*<0.05) (Table 1). These results indicate that the expression levels of MIR155HG and TNF-α can affect the activity of SLE.

**Table 1:** Comparisons of MIR155HG and TNF-α among patients with different SLEDAI scores. ^*^P<0.05, compared with SLEDAI 0–4 score; ^#^P<0.05, compared with SLEDAI 5–9 score; ^&^P<0.05, compared with SLEDAI 10–14 score.

SLEDAI	n	MIR155HG	TNF-α
0–4 score	66	1.54±0.57	1.88±0.67
5–9 score	35	2.25±1.02^*^	2.87±1.32^*^
10–14 score	19	2.82±1.24^*#^	4.01±2.03^*#^
≥15 score	10	3.54±1.69^*#&^	4.87±2.76^*#&^

### Correlation analysis of MIR155HG and TNF-α with demographic and immunological data of patients

There were 36 males and 94 females enrolled in this study, including 54 cases aged <55 years old and 76 cases aged 55 years old. There were no statistically significant differences in the levels of MIR155HG and TNF-α among patients with different genders and ages (*P*>0.05). Besides, dsDNA antibody, Sm antibody, U1-RNP antibody, AnuA and P-Prot antibody were detected, based on which the patients were grouped. The results of statistical analysis revealed that the levels of MIR155HG and TNF-α had no statistically significant differences among patients with dsDNA antibody, Sm antibody, U1-RNP antibody, AnuA or P-Prot antibody (*P*>0.05) (Table 2 and Table 3). The above findings demonstrate that the expressions of MIR155HG and TNF-α were balanced and comparable among the 130 patients with different ages, genders and dsDNA antibodies.

**Table 2:** Relationship between MIR155HG and Demographic and Immunological data. Note: dsDNA: Anti-double stranded DNA; Sm: Anti-Smith antibody; U1-RNP: Anti-U1 Ribonucleoprotein; AnuA: anti-nucleosome antibody; P-Prot: anti-ribosomal P protein antibody

Variable	n/percentage (%)	MIR155HG	*t*	*P *
Sex				
Male	36/27.–7	2.14±1.27	-1.319	0.190
Femal	94/72.3	2.65±2.18		
Age (years old)				
<55	54/41.5	2.77±1.01	0.908	0.366
≥ 55	76/58.5	2.58±1.23		
dsDNA				
Positive	62/47.7	3.14±1.86	1.176	0.242
Negative	68/52.3	2.79±1.53		
Sm				
Positive	38/28.2	1.89±0.94	-1.915	0.058
Negative	92/70.8	2.46±1.73		
U1-RNP				
Positive	58/46.6	2.13±1.76	-1.856	0.066
Negative	72/55.4	2.88±2.64		
AnuA				
Positive	39/30.0	2.73±1.33	-0.134	0.893
Negative	91/70.0	2.76±1.09		
P-Prot				
Positive	28/21.5	3.05±1.05	-1.560	0.322
Negative	102/78.5	2.69±1.83		

**Table 3:** Relationship between TNF-α and Demographic and Immunological data. Note: dsDNA: Anti-double stranded DNA; Sm: Anti-Smith antibody; U1-RNP: Anti-U1 Ribonucleoprotein; AnuA: anti-nucleosome antibody; P-Prot: anti-ribosomal P protein antibody

Variable	n/percentage (%)	TNF-α	*t*	*P*
Sex				
Male	36/27.7	3.11±1.43	-0.307	0.759
Female	94/72.3	3.20±1.52		
Age (years old)				
<55	54/41.5	3.57±1.83	1.162	0.247
≥55	76/58.5	3.25±1.31		
dsDNA				
Positive	62/47.7	2.75±1.08	0.423	0.673
Negative	68/52.3	2.66±1.32		
Sm				
Positive	38/28.2	2.94±1.51	-1.216	0.226
Negative	92/70.8	3.37±1.95		
U1-RNP				
Positive	58/46.6	2.65±1.32	-0.880	0.380
Negative	72/55.4	2.87±1.49		
AnuA				
Positive	39/30.0	2.71±1.45	-1.857	0.066
Negative	91/70.0	3.52±2.55		
P-Prot				
Positive	28/21.5	2.88±1.65	0.325	0.746
Negative	102/78.5	2.75±2.25		

### Correlation analysis of MIR155HG and TNF-α with complications during treatment

Among the 130 patients with SLE, there were 10 cases of infection, 7 cases of blood system damage, 37 cases of renal damage, 28 cases of central nervous system damage, 11 cases of pulmonary damage, and 30 cases of cardiac damage during treatment. To explore whether MIR155HG and TNF-α are associated with these complications, Spearman correlation analysis was performed. The results showed that MIR155HG and TNF-α were positively correlated with the incidence of infection, renal damage and cardiac damage (*r*=0.623, 0.533 and 0.621.; *r*=0.431, 0.498 and 0.552) (*P*<0.05), but had no obvious correlations with the incidence of blood system damage, central nervous system damage and pulmonary damage (*P*>0.05) (Table 4). The above results suggest that the expressions of MIR155HG and TNF-α have positive correlations with the incidence of infection, renal damage and cardiac damage during treatment. The higher the expressions of MIR155HG (r=0.623; P<0.05) and TNF-α (r=0.431; P<0.05) are, the higher the incidence rates of infection, renal damage and cardiac damage will be. 

**Table 4:** Relationships between MIR155HG/TNF-α and complications. Note: *P* value <0.05 is considered statistically significant.

Complications	n/percentage (%)	MIR155HG		TNF-α	
		r	P	r	P
Infections					
No	120/92.3	0.623	0.007	0.431	0.025
Yes	10/7.7				
Blood system damage					
No	123/94.6	0.076	0.173	0.397	0.286
Yes	7/5.4				
Renal damage					
No	93/71.5	0.533	0.018	0.498	0.003
Yes	37/28.5				
Central nervous system					
No	102/78.5	0.321	0.374	0.284	0.411
Yes	28/21.5				
Lung injury					
No	119/91.5	0.287	0.052	0.142	0.177
Yes	11/8.5				
Cardiac damage					
No	100/76.9	0.621	<0.001	0.552	0.004
Yes	30/23.1				

### Association between MIR155HG and TNF-α

**Figure 1:**
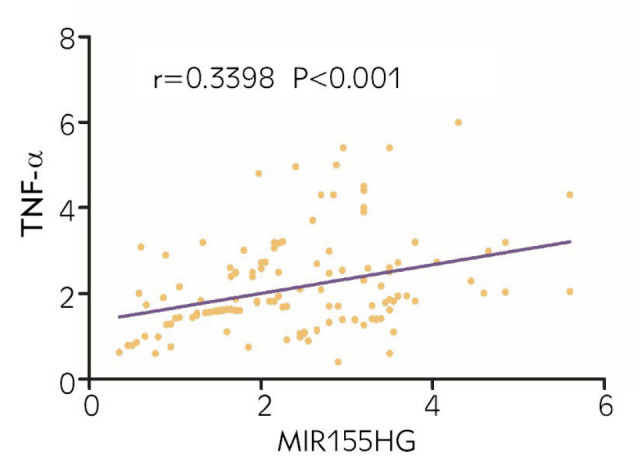
Association between MIR155HG and TNF-α. There was a positive correlation between the expressions of MIR155HG and TNF-α (r=0.3398, P<0.001).*P < 0.05.

To clarify whether there is an association between the expressions of serum MIR155HG and TNF-α in patients, Pearson correlation analysis was performed. The results manifested that the expressions of MIR155HG and TNF-α were positively correlated with each other (*r*=0.3398, *P*<0.001) (Figure 1). It can be seen that MIR155HG can positively regulate the expression of TNF-α, affecting the efficacy on patients.

### Analysis of risk factors for prognosis of SLE patients

A total of 130 patients were followed up, resulting in 15 cases of death (11.54%) within 5 years, with an average follow-up duration of 4.89 years. Based on the mean (2.51; 3.18) expressions of MIR155HG and TNF-α, the patients were divided into high expression group and low expression group. The results of COX regression analysis manifested that the patient's gender, age and SLEDAI score had no remarkable correlations with the prognosis (*P*>0.05). Highly-expressed MIR155HG and TNF-α, and complications during hospitalization (infection, blood system damage, renal damage, central nervous system damage, pulmonary damage and cardiac damage) were the risk factors for prognosis of patients (Table 5). These results confirm that high expressions of MIR155HG and TNF-α are harmful to the prognosis of patients.

**Table 5:** Related factors of prognosis. Note: *P* value <0.05 is considered statistically significant.

Variable	HR	95% *CI*	*P*
Male	1.317	0.249–1.615	0.086
Age (≥55 years old)	0.791	0.462–2.314	0.074
SLEDAI ≥15	1.248	0.775-–3.204	0.145
Complication infection	1.441	1.150–1.753	0.011
Blood system damage	1.753	1.324–3.041	0.037
Renal damage	1.852	1.520–2.753	0.005
Central nervous system damage	2.149	1.693–4.337	<0.001
Lung injury	1.433	1.185–2.074	0.028
Cardiac damage	2.627	1.465–4.194	0.006
MIR155HG (High level)	1.973	1.284–3.441	0.008
TNF-α (High level)	2.143	1.663–4.321	<0.001

## Discussion

Patients with SLE suffer from abnormal secretion of various cytokines, and these cytokines are interrelated and promote each other, leading to cytokine network disorders, immune disorders and multiple organ damage [14]. In recent years, scholars in China and foreign countries have explored the pathogenesis of SLE from the basic level to the clinical level, from the environmental infection to the individual, and from the whole to the cell, protein, gene and epigenetic modification. Despite great achievements made in the understanding of SLE, there is still a lack of breakthrough, and its specific pathogenesis remains unclear [15] [16].

LncRNAs exert important functions in cellular life activities through a variety of mechanisms, such as epigenetic modification, post-transcriptional processing, regulation and translation [17]. LncRNAs are also associated with cell differentiation and activation, and play a key role in regulating the differentiation and activation of immune cells in the innate and acquired immune system, thereby affecting the occurrence and development of SLE [18] [19].The research on the mechanism of lncRNAs in the occurrence and development of SLE can lay a foundation for seeking markers for early diagnosis, drugs and biotherapy targets.

MIR155HG contains the precursor sequence of miR-155. The role of miR-155 from MIR155HG in SLE has been reported in the literature [20]. MiR-155 is highly expressed in the urine of SLE patients, and its expression is related to proteinuria and SLEDAI [21]. In this study, it was found that the expression of MIR155HG was related to SLEDAI. With the increase of activity, the expression of MIR155HG also markedly rose. 

TNF-α is synthesized by a variety of cells, including T and B lymphocytes and activated mononuclear macrophages. Suarez A. et al [22] studied and found that the interaction between IL-10 and TNF-α can affect the susceptibility of patients to SLE and the formation of specific autoantibodies, and highlyexpressed TNF-α is an important risk factor for patients with SLE. In 2004, Aringer M. et al [23] treated six patients with systemic lupus erythematosus (SLE) using TNF-α inhibitors. They observed improvements in the patients' inflammatory responses and clinical manifestations, suggesting that anti-TNF-α therapy could alleviate local tissue damage in SLE patients. However, it is also reported [24] that the level of TNF-α in patients with SLE in remission is higher than that in patients with active SLE and normal people. It is believed that TNF-α may also be a protective cytokine for SLE. Since the research results about the role of TNF-α in the pathogenesis of SLE are inconsistent, further research is still needed. In this study, it was found that the level of serum TNF-α in SLE patients was positively correlated with the severity of disease. In addition, according to correlation analysis, the expressions of miR-155 and TNF-α are positively correlated with each other in the urine of SLE patients, which may play an important role in the pathophysiology of SLE [21]. Similarly, in this study, the results revealed that the level of serum TNF-α in SLE patients rose with the increase of SLEDAI score, and the expression of TNF-α was positively correlated with MIR155HG. MIR155HG and TNF-α jointly affected the occurrence of complications and the prognosis of SLE. Highly-expressed MIR155HG and TNF-α would promote the occurrence of such complications as infection, renal damage and cardiac damage in SLE patients. Therefore, MIR155HG and TNF-α were the risk factors for the prognosis of patients.

## Conclusion

MIR155HG and TNF-α affect the activity of SLE, and the high expressions of them promote the occurrence of such complications as infection, renal damage and cardiac damage, harming the prognosis.

## Dodatak

### Conflict of interest statement

All the authors declare that they have no conflict of interest in this work.
